# Comparison of Surgical Wait Times and Procedure Length in the Management of Postaxial Polydactyly Using Local or General Anesthesia

**DOI:** 10.1177/22925503221134813

**Published:** 2022-11-17

**Authors:** Adam Mosa, Mithila Somasundaram, Diba Vahidi Ferdosi, Kristen Davidge, Howard M. Clarke, Emily S. Ho, Terence Kwan-Wong

**Affiliations:** 1 Division of Plastic and Reconstructive Surgery, 7979 The Hospital for Sick Children, Toronto, Canada; 2 Temerty Faculty of Medicine, 7938 University of Toronto, Toronto, Canada; 3 Department of Occupational Science and Occupational Therapy, 7938 University of Toronto, Toronto, Canada

**Keywords:** ulnar polydactyly, surgical wait time, local anesthesia, pediatric hand surgery, anesthésie locale, opération de la main en pédiatrie, polydactylie cubitale, temps d’attente en chirurgie

## Abstract

**Introduction:** For infants with ulnar polydactyly, surgical removal of the supernumerary digit can be performed under general or local anesthetic. This study evaluated the wait times, surgical duration, and sedation times associated with performing the procedure under local versus general anesthetic in infants with ulnar polydactyly. **Methods:** The databases of three surgeons at our institution were reviewed for children less than 2 years of age who underwent surgery for non-syndromic ulnar polydactyly. Data collection included patient demographics, wait times, duration of surgery and sedation and complications. **Results:** The study included children (n  =  55) who received treatment under local (n  =  22) or general (n  =  33) anesthesia. The wait times for the local anesthetic group were significantly shorter than the general anesthetic group (p < 0.05) for: referral to first consultation appointment; referral to surgery date, and decision date to surgery date. The duration of surgery (17.9  ±  6.9 vs 36.6  ±  20.2 min) and sedation time (26.3  ±  11.1 vs 74.8  ±  29.1 min) were significantly shorter in the local anaesthetic group (p < 0.05). There were no differences in complication rates between the groups. **Conclusion:** In this single-institution retrospective analysis, treatment of non-syndromic ulnar polydactyly with local anesthetic and bottle sedation was associated with shorter wait times, and duration of surgery and sedation. **Level of Evidence:** III, retrospective chart review and quality improvement initiative

## Résumé

**Introduction :** Chez les nourrissons présentant une polydactylie cubitale, il est possible de procéder à l’ablation chirurgicale du doigt excédentaire sous anesthésie locale ou générale. La présente étude visait à évaluer les temps d’attente, la durée de l’opération et la durée de la sédation associés à l’intervention lorsqu’elle était effectuée sous anesthésie locale ou générale chez les nourrissons présentant une polydactylie cubitale. **méthodes :** Les chercheurs ont fouillé les bases de données de trois chirurgiens de leur établissement pour en extraire les cas d’enfants de moins de deux ans ayant subi une opération de la polydactylie cubitale non syndromique. La collecte de données incluait les caractéristiques des patients, les temps d’attente, la durée de l’opération et de la sédation et les complications. **Résultats :** La présente étude incluait des enfants (n=55) qui avaient été traités sous anesthésie locale (n=22) ou générale (n=33). Le temps d’attente du groupe sous anesthésie locale était beaucoup plus court que celui du groupe sous anesthésie générale (p<0,05) pour ce qui est de la période entre l’orientation et la première consultation, l’orientation et la date de l’opération, ainsi que la date de la décision et la date de l’opération. La durée de l’opération (17,9 ± 6,9 minutes par rapport à 36,6 ± 20,2 minutes) et de la sédation (26,3 ± 11,1 minutes par rapport à 74,8 ± 29,1 minutes) était beaucoup plus courte dans le groupe sous anesthésie locale (p<0,05). Il n’y avait pas de différence entre les groupes à l’égard des taux de complications. **Conclusion :** Dans cette analyse rétrospective mono-institutionnelle, le traitement de la polydactylie cubitale non syndromique accompagné d’une anesthésie locale et d’une sédation par bonbonne était associé à une diminution du temps d’attente ainsi que de la durée d’opération et de sédation.

## Introduction

Ulnar polydactyly is one of the most common congenital hand diagnoses in pediatric plastic surgery and constitutes a large number of new patient referrals to our center annually. Reducing wait times for patients with ulnar polydactyly depends on speed of triage from initial referral to consultation as well as time from consultation to surgical management. Establishing safe and effective ways to mitigate growing waitlists has become increasingly important.

Polydactyly is a condition characterized by the presence of one or more supernumerary digits on an extremity.^1^ Children with postaxial (ulnar) polydactyly present with a supernumerary digit on the ulnar side of the hand which can be classified into two subtypes: Type A and Type B.^2,3^ Type A refers to well-developed digits with a bony articulation against either a duplicated metacarpal or the fifth digit metacarpal.^3^ Type B describes a digit that is small, poorly formed, and connected to the fifth digit via a soft tissue pedicle.^3^ Surgical treatment of ulnar polydactyly may be performed for a variety of reasons including functional, cosmetic, and psychological factors.^2,4^ Surgical removal of the extra digit in ulnar polydactyly can be performed under local anesthetic shortly after birth,^5^ or under general anesthetic (GA) at around six to twelve months of age.^3^ The older age of GA excision is chosen to reduce associated surgical risks such as airway complications, cardiac arrest, and the potential neurocognitive impact of GA in infancy.^6^

At our institution, surgical treatment has been traditionally performed at 6 to 12 months of age under general anesthetic. A new care model was introduced using local anesthetic and bottle sedation in an ambulatory setting for children 3 months of age and younger. The primary aim of this study was to evaluate the wait times and procedure durations with management of infants presenting with postaxial polydactyly Type B under general anesthesia compared to local anesthesia. The identification of management differences would inform practice to promote greater cost efficiency, patient throughput and allow earlier treatment.

## Materials and Methods

### Patient Selection

Using a retrospective study design, this single-institution chart reviewed the surgical databases of three surgeons. Inclusion criteria included: children with non-syndromic unilateral or bilateral Type B ulnar polydactyly; and procedures performed from 2016 to 2019 under local or general anesthesia. All ulnar polydactyly excisions performed in children less than 2 years of age were collected for analysis.

Data collection included: patient demographics; time from referral to consultation; time from consultation to surgery; duration of surgery, duration of sedation; and complications. Sedation duration for children undergoing general anesthetic was defined as the time from administration of the anesthetic medicine to the end of procedure time. Patient records were reviewed to identify any postoperative complications including infection, bleeding, return to the emergency department, or anesthetic related issues. This study was approved by the Research Ethics Board (REB Number: 1000065499). Informed consent was obtained from all individual participants included in the study.

### Surgical Procedure

Local anesthetic procedures were performed in the ambulatory clinic. A bipolar cautery machine was brought to clinic for these procedures and a sterile basic surgical instrument tray was obtained including 15-blade scalpel, dissecting scissors, fine-tooth forceps, and needle driver. The protocol involved parent administered formula or breast milk feeding followed by oral sucrose solution 5-10 min prior to procedure start time. Sedation start time was defined as the time feeding was initiated. Once the child was fed, the primary surgeon administered local anesthetic to the base of the duplicated digit using 0.5 cc of 1% lidocaine with epinephrine (1:100 000), buffered with sodium bicarbonate. Careful dosing is calculated to avoid risk of local anesthetic toxicity and no more than 7 mg/kg per dose of lidocaine with epinephrine is administered. The patient was swaddled and positioned securely in the parent's arms with both child and parent lying supine on a stretcher. Under sterile conditions, a primary surgeon and secondary assistant position and stabilize the hand. A clinic nurse was available for additional support as needed. After confirming anesthetic effect and using 2.5-3.5× loupe magnification, an elliptical incision was made at the base of the digit. The neurovascular bundles were visualized to allow precise identification and high transection of the accessory digital nerve. Bipolar cautery was used to seal the accessory digital artery. The wound was closed with 5-0 absorbable sutures and skin adhesive glue applied as a waterproof barrier. No additional dressings were applied.

The general anesthetic group followed a similar operative protocol, without being swaddled or a bottle feeding. These children had general anesthetic induction with placement of laryngeal mask airway. Following the procedure, they were transferred to recovery and reunited with parents.

There was no post-operative immobilization protocol or activity restriction in either group.

### Data Analysis

Data were summarized using means and standard deviations. The Shapiro-Wilk test of normality was significant; therefore, comparative analyses were performed using a Mann-Whitney U test. Patient demographics were compared using a Chi-Square analysis. The significance level was set to 0.05. Data analysis was performed using IBM^©^ SPSS^©^ Version 25.

## Results

**Figure 1.**
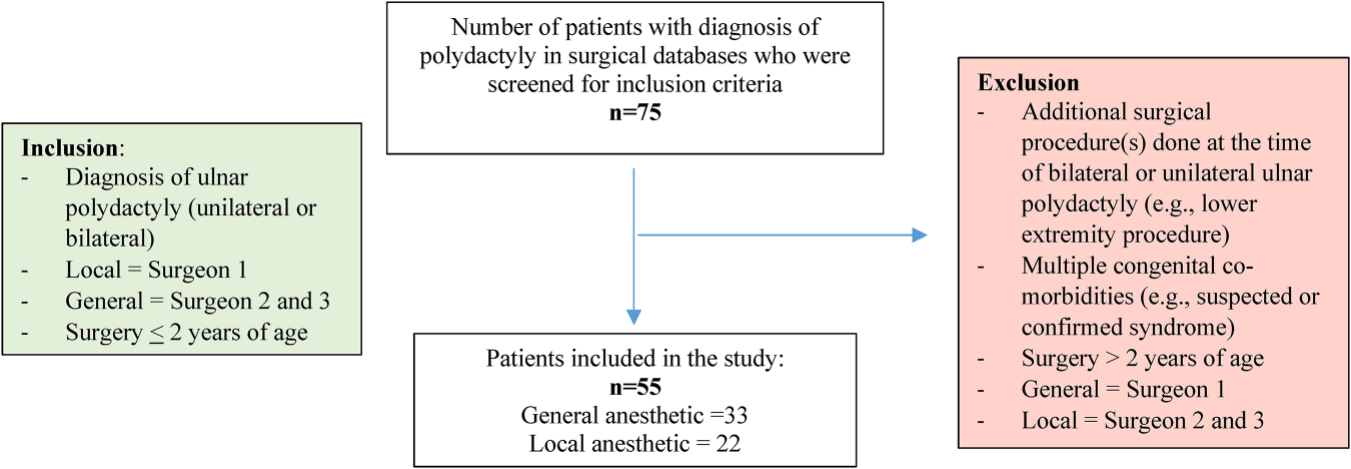
Selection criteria flow diagram.

A total of 75 medical records were reviewed, and 55 patients met the inclusion criteria (Figure 1). Twenty-two patients (40%) underwent supernumerary digit removal under local anesthetic and 33 patients (60%) had general anesthetic (Table 1). Sex distribution and laterality were similar between the two groups. The mean age at referral of the local anesthetic group was significantly (p < 0.05) lower than the general anesthetic group (16.3  ±  21.6 days vs 73.9  ±  106.1 days, respectively).

**Table 1.** Patient Demographics.

	Total Sample (n = 55) N	Local Anesthetic (n = 22) N	General Anesthetic (n = 33) N
Sex			
Females	20	5	15
Males	35	17	18
Hand Involvement			
Bilateral	42	16	26
Unilateral	13	6	7
Mean Age at Referral (Days)	44.7	16.3 Range 0–77	73.9 Range 0–465

The wait times from referral to initial consultation and from referral to surgery were significantly shorter (p < 0.05) in the local anesthetic group compared with the general anesthetic group (Table 2). Similarly, the duration of time between the decision date and surgery was significantly shorter (p < 0.05) for the local anesthetic group compared to the general anesthetic group (19.0  ±  14.2 days: range 1-43 days vs 131.4  ±  113.8 days: range 16-647 days) (Table 2). The general anesthetic cohort had outlier values in the data set (eg 647 days from decision for surgery to operative date), but removing these samples did not impact the statistical significance between groups. Unfortunately, case specific explanations for the delays are not captured in the data set. These values contribute to the larger standard deviation in GA group.

**Table 2.** Wait times, surgery duration, and sedation duration in the local and general anesthetic procedures.

	Local Anesthetic			General Anesthetic		
	Mean ± standard deviation	Minimum	Maximum	Mean ± standard deviation	Minimum	Maximum
Referral date to first clinic appointment date (days)	21.2 ± 18.0	1	71	111.4 ± 66.9	1	242
Referral date to surgery date (days)	40.2 ± 21.1	12	85	243.0 ± 117.3	99	652
Decision date to surgery date (days)	19.0 ± 14.2	1	43	131.4 ± 113.8	16	647
Surgery duration (minutes)	17.9 ± 6.9	8	30	36.6 ± 20.2	9	93
Sedation duration (minutes)	26.3 ± 11.1	8	45	74.8 ± 29.1	27	147

The duration of surgery and duration of sedation were significantly shorter (p < 0.05) in the local anesthetic group (17.9  ±  6.9 min: range 8-30 min and 26.3  ±  11.1 min: range 8-45 min, respectively) compared with the general anesthetic group (36.6  ±  20.2 min: range 9-93 min and 74.8  ±  29.1 min: range 27-147 min, respectively) (Table 2). There were no postoperative complications reported.

## Discussion

This study demonstrated that supernumerary digit removal under local anesthetic was associated with a significant reduction in wait times, surgical duration, and sedation time compared to general anesthetic. There is limited patient and parental reported outcomes in the existing literature. A systematic review by Samarendra et al highlights that treatment-delay related family distress and dissatisfaction associated with the stigma of accessory digits may be alleviated via more streamlined systems for surgical management.^7^ The shorter wait times achieved in our study provide the opportunity for earlier intervention which may reduce parental anxiety and the negative consequences associated with perceived or actual stigma. Further research into parental and patient reported outcomes is needed.

Previously described methods using suture ligation and surgical clip application for children under 6 months of age allow for earlier intervention but are associated with complications such as residual soft tissue deformity, scar hypersensitivity and neuroma formation leading to pain at the amputation site.^4,8,9^ Operative treatment has the advantage of anatomic dissection and amputation of the digital neurovascular bundle under direct visualization, with the lower risk of neuroma formation. This study demonstrated that surgical removal of the supernumerary digit in ulnar polydactyly using local anesthetic has a low early post-operative complication rate.

A previous retrospective study evaluated the costs associated with performing supernumerary digit removal in infants with ulnar polydactyly under local and general anesthetic.^10^ The study demonstrated that costs of the procedure using general anesthetic was on average $3063.94 USD more than local anesthetic and with every additional minute of operating time costing an additional $71.86 USD.^10^ Length of post-operative stay and operating room time were some of the factors associated with the increase in costs in the general anesthetic group.^10^ In our institution, the local anesthetic procedure has transitioned to the clinic/procedure room setting and is no longer performed in the main operating room. This eliminates the need for operating room access and reduces costs through less staffing and sterile supply requirements.

One concern associated with local anesthetic use is that the child is not immobilized as they would be under general anesthesia. Our results found no reported complications in both groups. The external methods of patient immobilization used in our study (positioning that limits arm movement) as well as soothing with formula feeding or breastfeeding prior to the procedure are effective in ensuring stabilization of the patient. Children are also provided with pre-operative sucrose solution for soothing and analgesia.

One limitation of this study was the lack of data on the experiences of patient families. Factors such as parent preference and level of satisfaction with post-operative results are relevant considerations that can influence the type of anesthetic used for the procedure. Future studies can also consider performing a comparative analysis of the costs, long-term post-operative outcomes, and rate of revision surgeries associated with performing supernumerary digit removal using general anesthetic versus local anesthetic. This would provide greater insight of each method, allowing surgeons to provide more information to parents who are deciding on the most appropriate treatment option for their child.

The results of our study are clinically relevant and can be translated to several aspects of clinical practice and hospital management. For example, educating referring physicians about these findings can allow for earlier referrals and consequently, earlier intervention. This study supports transitioning future cases to a minor procedure setting and allows operating room time to be diverted to other needed operative procedures. COVID-19 has further contributed to long surgical wait times and provides the impetus to seek quality improvement measures that improve efficiency. Interventions that increase patient throughput by utilizing ambulatory resources will help to address both the pre-existing wait times and anticipated back logs as the Canadian healthcare system navigates the post-COVID19 period.

This single-institution retrospective analysis demonstrated that local anesthetic use was associated with shorter wait times, surgery times, and sedation times than general anesthetic use in the surgical treatment of ulnar polydactyly in infants. Future studies should incorporate parental input, as well as a multi-institution cost analysis comparing costs associated with performing the procedure using each anesthetic type, to help inform decision-making when assessing infants with ulnar polydactyly.
